# Efficacy, Safety, and Tolerance of a New Injection Technique for High- and Low-Molecular-Weight Hyaluronic Acid Hybrid Complexes

**Published:** 2015-10-08

**Authors:** Carmen Laurino, Beniamino Palmieri, Alessandro Coacci

**Affiliations:** ^a^Department of General Surgery and Surgical Specialties, University of Modena and Reggio Emilia Medical School, Surgical Clinic, Modena, Italy; ^b^Department of Surgical Urgency and First Aid, USL Grosseto, Grosseto, Italy

**Keywords:** high and low molecular weight, hyaluronic acid, hydration, elasticity, TEWL

## Abstract

**Objective:** Facial aging is characterized by skin laxity and loss of skin elasticity. Hyaluronic acid, a biological component of the extracellular matrix, whose level decreases during aging, plays structural, rheological, and physiological roles in the skin. Hyaluronic acid may possess different molecular weights: low-molecular-weight hyaluronic acid (from 50 kDa) and high-molecular-weight hyaluronic acid (just up to 2 million kDa). This monocentric, retrospective, observational study investigates the efficacy, security, and tolerability of a new injective low- and high-molecular-weight hyaluronic acid for facial skin rejuvenation. **Methods**: Eleven women received once a month, for 2 months, 2 mL of the product in the subcutaneous layer of the right and left malar/submalar areas. Facial skin echography, facial skin hydration, elasticity, and transepidermal water loss were assessed before (*T*_0_), after 1 month (*T*_1_), and after 3 months of treatment (*T*_2_). The injective features of the product, physician subjective satisfaction, and patient satisfaction were also reported. **Results**: Facial face hydration, elasticity, and transepidermal water loss values significantly improved at *T*_1_ and *T*_2_ (*P* < .01). Patients were very satisfied at the end of the treatment, and the compound's profit evaluated by the physician was optimal in the absence of local side effects. **Conclusions**: This treatment represents a good treatment option to restore vitality and turgidity of skin presenting the signs of aging in the absence of intolerance symptoms.

Reduced dermis vascularization and biosynthesis of extracellular matrix by fibroblasts, with subsequent skin laxity and elastic properties loss, are common features of facial aging.^1^ Hyaluronic acid (HA), a glycosaminoglycan (GAG) binding water molecules and involved in tissue hydration, is markedly reduced in the aged skin.^2^^,^^3^ This molecule and its complexes have structural, rheological, physiological, and cell-to-cell functions in the skin: it promotes the fibroblast proliferation and migration, acts as a free radical scavenger, and, due to its optimal water-binding capacity, improves nutrient exchange between vessels and the dermis.^4^ Its structure is not species-specific and it is highly biocompatible.^5^ More than half of the HA in our body is distributed in the cutaneous region where fibroblasts, keratinocytes, and endothelial cells of the dermal microcirculation synthesize families of HA ranging from 50 kDa (low molecular weight, L-HA) to 2 million kDa (high molecular weight, H-HA).^6^ Once the varying classes of HA have been synthesized, they operate in harmony with the different cutaneous compartments, where they interact with specific receptors (CD44, hyaluronan-mediated motility receptors [RHAMM], Lyve-1), triggering different cell responses and biochemical cascades.^7^

In the epidermis, HA, owing to its high negative charge, is localized in the extracellular region, where it forms a hydrophilic network transporting and distributing nutrients and metabolites to the keratinocytes, melanocytes, and the Langerhans cells through a percolation mechanism, establishing important functional interactions.^6^ This prevalent, but not exclusive, trophometabolic activity of L-HA contributes to the maintenance of the cutaneous homeostasis. In the same way, in the dermis, H-HA, through the complex interactions with the main molecules of the extracellular matrix (proteoglycans, glycoproteins, elastin, and the 7 different types of collagen), plays a pivotal role in the maintenance of structural stability.^8^ HA works like a physiological skin expander, occupies the micro domains of the dermal matrix, cancels its depressions, and gives the cutis a smooth and polished macroscopic appearance.^9^

In summary, the “trophic-modeling” action of HA arises from its synergy and the activity connected with its molecular weights. L-HA can be either produced by direct synthesis or self-generated by postsynthesis reduction (H-HA to L-HA transition), carried out by specific enzyme systems following modifications of physiological homeostasis. Increasing knowledge of the functions performed by the different molecular weights has stimulated the need to be able to provide HA in the derma-aesthetic field, using different molecular weights and concentrations.

The physiological reduction of HA activities in the aged skin contributes to dermal thinning and the formation of folds and wrinkles.^4^ The procedure of HA injections in the dermis, defined “viscosupplementation,” restores the skin tone, fullness, and elasticity, although native HA remains stable in the dermis only for few days due to the quick degradation by hyaluronidase.^10^ For this reason, chemically stabilized cross-linked HA filler has been developed to prolong dermal stability.^4^ Although 1,4-butanediol diglycidyl ether is the safest and the most used cross-linking agent, the modern filler technologies aim to reduce the concentration of this chemical compound to increase filler biocompatibility.^11^

The development of increasingly sophisticated synthesis technologies has recently led IBSA Farmaceutici Italia Srl to provide a whole range of HAs at defined concentrations and molecular weights. In particular, the production of novel, stable, molecular hybrids proved feasible through a specific procedure involving a thermal treatment. For the first time, it was possible to combine 32 mg of H-HA (1100–1400 kDa) and 32 mg of L-HA (80–100 kDa) to obtain injectable concentrations of HA unimaginable until now (64 mg in 2 mL). The H-HA–L-HA complex (1:1 weight ratio) was produced following the procedure described in patent application WO2011EP65633.^12^ This new product is called Profhilo. Profhilo hybrid complexes proved to protect the H-HA present, reducing 8-fold degradation of HA chains of molecular weight of more than 1000 kDa. Moreover, L-HA is slowly released from the hybrid network; therefore, the first inflammatory cytokines are not upregulated, lowering overall the inflammatory cascade, or limiting the first “inflammation” phase to a positive light shock to “sleeping/tired” cells.

The aim of this monocentric, retrospective, observational study was to investigate the efficacy, security, and tolerability of a new medical device for facial skin rejuvenation.

## METHODS

### Tested product

3.2% of 32 mg (H-HA) + 32 mg (L-HA)/2 mL of HA sodium salt produced following the procedure described in patent application WO2011EP65633 and distributed by IBSA Farmaceutici Italia Srl.

### Clinical study

Retrospective, monocentric, observational study.

### Patients

Eleven women aged 48 to 67 years (mean = 56 years) participated in this study. Inclusion criteria were moderate facial photoaging, as determined by the visual analog scale (VAS; VAS score ˂4 = low degree of photoaging; 4 > VAS score ˂ 7 = moderate degree of photoaging; VAS score >7 = high degree of photoaging). Exclusion criteria were skin allergies, facial dermatitis, precancerous facial skin lesions due to prolonged ultraviolet (UV) exposure, autoimmune diseases, and permanent filler implant in the treated area.

### Treatment

The procedure was performed at controlled environmental conditions (21°C; 30%–40% relative humidity). A 2 mL of the product was injected in the subcutaneous layer of the right and left malar/submalar areas. Five injection sites for each side were identified with an acupuncture point finder (Point Mate, Acu-Locator detector; ib3 health, Vancouver, British Columbia, Canada). This probe allows identification of lower impedance points. The instrument detects relatively avascular subdermal areas able to spread HA along the interstitial spaces between lymphatic vessels.^13^ The product was administered with a 29-G needle using a bolus technique. Two sessions were performed, once a month, for 2 months.

### Objective evaluations

A DermaLab USB Single Parameter Units (Hadsund, Denmark) was used to analyze facial hydration, facial elasticity expressed as retraction of extended skin (Young's modulus), and facial transepidermal water loss (TEWL) pre- and posttreatment. The modulus of longitudinal elasticity—Young's modulus—defines the relation between stress (σ) and strain (ε) in the skin. The modulus characterizes skin resistance to elastic elongation.^14^ Hydration was expressed by conductance principle in micro-Siemens units (µS), elasticity was expressed by stress/strain using the applied vacuum principle in megapascals (MPa), and TEWL was expressed by diffusion gradient in grams per meter square per hour (g/m^2^/h). An ultrasound probe Esaote LA 435 (an 18-MHz convex probe weighing 250 g) was used to analyze ultrasound signals pre- and posttreatment. This instrument produces an ultrasound-emitted beam that is reflected by the dermal tissues according to its stromal density and vascular tone. The probe was applied perpendicularly over the facial skin without any further pressure to evaluate the soft tissues ultrasound morphology. The skin parameters for clinical evaluation were assessed before (*T*_0_), after 1 month (*T*_1_), and after 3 months of treatment (*T*_2_). Photographs of the patients were collected at the baseline visit and at each follow-up visit (*T*_0_, *T*_1_, and *T*_2_). Adverse events were monitored during the entire study period.

### Subjective evaluations

The physician evaluated injective features of the product as follows: (*a*) easy to inject; (*b*) acceptable; (*c*) a little acceptable; and (*d*) not acceptable. Physician subjective satisfaction was assessed at each follow-up visit as follows: (*a*) optimal; (*b*) good; (*c*) satisfactory; (*d*) poor; and (*e*) inefficient. Patient satisfaction was assessed as follows: (*a*) very satisfied; (*b*) satisfied; and (*c*) not satisfied.

### Statistical analysis

The instrumental data are presented as the mean ± SEM (standard error of the mean). An unpaired 2-sample Student *t* test was used to compare differences in skin parameters. All statistical analyses were performed with GraphPad Prism 6 (GraphPad Software Inc, San Diego, Calif). A value of *P* < .01 was considered significant.

## RESULTS

### Visual evaluations

**Figure 1:**
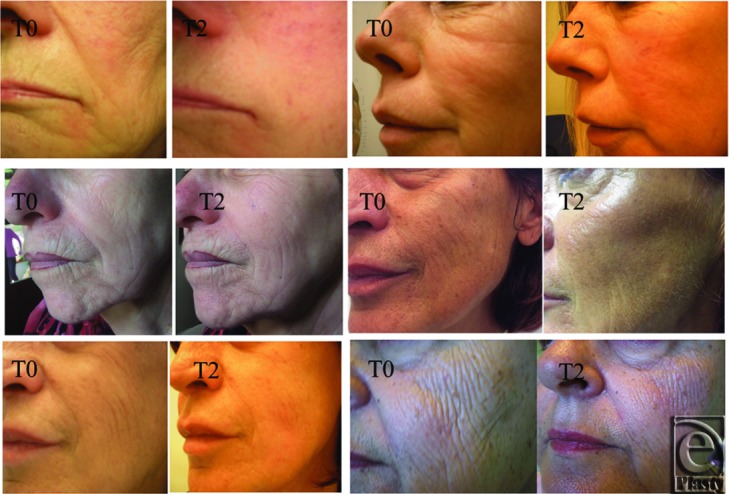
Photographs of the treated area before the treatment (*T*_0_) and 1 month after the second treatment (*T*_2_).

Visual pre- and postcomparison of this facial rejuvenation technique showed an improvement in skin wrinkles. At the end of the treatment (*T*_2_), facial skin appeared more bright and turgescent than before (*T*_0_) (Fig 1).

**Figure 2:**
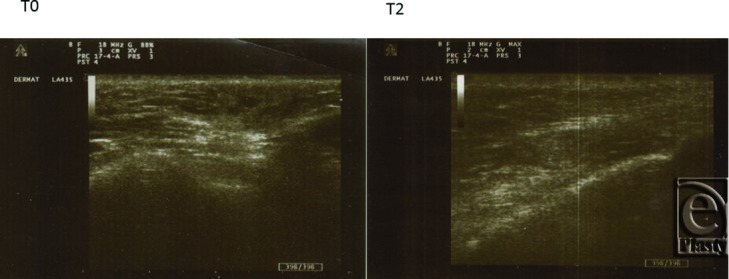
Malar area. *T*_0_: Evident widening of the deep dermal thickness, which is nonhomogeneous, with multiple echo scattering. *T*_2_: In this area, the collagen volume is not amplified, but the tissue hydration renders homogeneous the background, with reduced ultrasound beam reflection.

**Figure 3:**
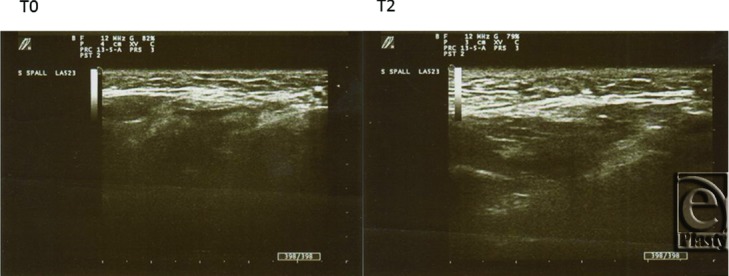
Submalar area. *T*_0_: Note the dermis subdermal layer. *T*_2_: Substantial widening of the subdermal area with increased echoes and well-arranged strands of collagen bundles newly formed after 3 months.

**Figure 4:**
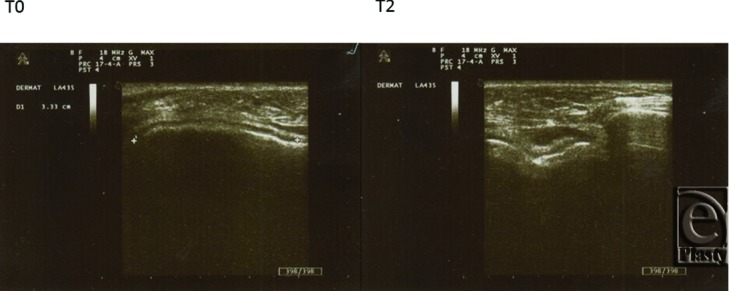
Zygomatic arch. *T*_0_: The convex subdermal profile is filled with multiple echoes over the bone ridge profile. *T*_2_: Newly induced collagen bundles induce denser echo reflexes.

Also changes in the echographic signals pre- and posttreatment have been observed. The examples are reported in (Figs 2–4).

### Objective evaluations

#### Hydration

**Figure 5:**
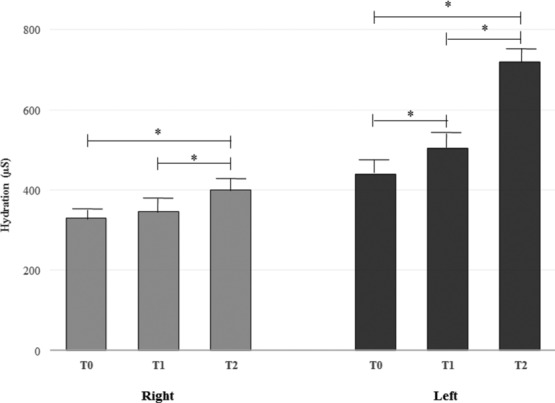
Facial skin hydration mean ± SEM values (^*^P < .01).

Facial skin hydration mean ± SEM values are reported in Table 1 and Figure 5. The data showed a significant improvement (*P* < .01) in hydration at *T*_1_ and *T*_2_ versus at baseline (*T*_0_) both for the right and left sides of the face.

#### Elasticity (Young's modulus)

**Figure 6:**
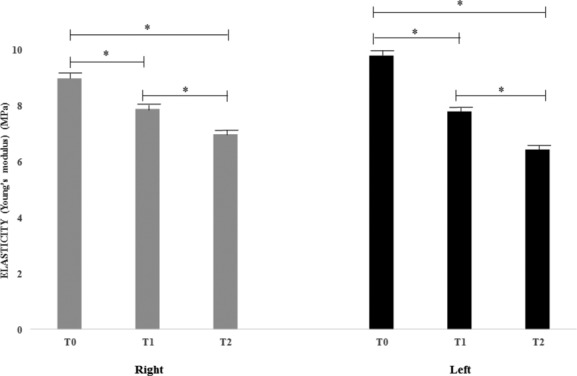
Facial skin elasticity (Young's modulus) mean ± SEM values (^*^P < .01).

Skin elasticity (Young's modulus) mean ± SEM values are reported in Table 2 and Figure 6. The data showed a significant improvement (*P* < .01) in elasticity at *T*_1_ and *T*_2_ versus at baseline (*T*_0_) both for the right and left sides of the face.

#### TEWL

**Figure 7:**
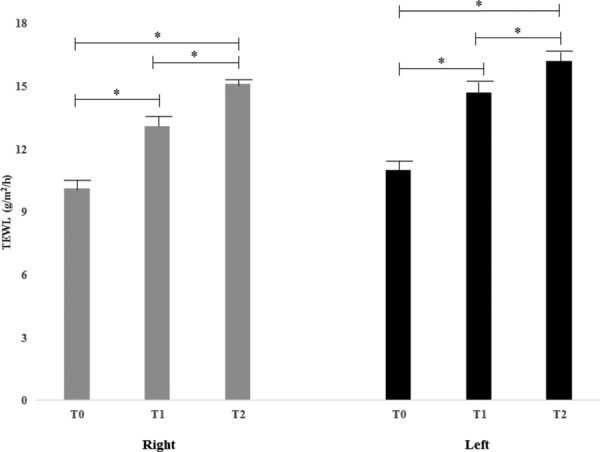
Facial skin TEWL mean ± SEM values (^*^P < .01). TEWL indicates transepidermal water loss.

Skin TEWL mean values are reported in Table 3 and Figure 7. The data showed a significant improvement (*P* < .01) in TEWL at *T*_1_ and *T*_2_ versus at baseline (*T*_0_) both for the right and left sides of the face.

### Subjective evaluations

Results of injective features of the product evaluated by the physician indicated that Profhilo was easy to inject (*a*) in 72.7% of cases (24/33) and acceptable (*b*) in 27.3% of cases (9/33). In no case (0/33), the injectability was declared little acceptable (*c*) or not acceptable (*d*).

Subjective assessment of effectiveness by the physician in the follow-up visits showed that benefits were optimal (*a*) in 51.5% of cases (17/33), good (*b*) in 45.5% of cases (15/33), and satisfactory (*c*) in 3.0% of cases (1/33). In no case (0/33), the effectiveness was reported poor (*d*) or inefficient (*e*). Results of patient satisfaction showed that in 87.9% of cases (29/33), women were very satisfied (*a*), and in 12.1% of cases, they were satisfied (4/33). A negative satisfaction (*c*) was not reported (0/33).

No adverse events were reported during the evaluation period except for mild side effects, such as localized hematomas appeared in 12.1% of total injections (4/33), which disappeared after 2 to 3 days.

## DISCUSSION

In the current evaluation, we have demonstrated efficacy, safety, and tolerance of a new skin rejuvenation procedure with high- and low-molecular-weight HA hybrid complexes injected into the lower impedance subdermal facial areas.

The loss of hydration, fine wrinkles, roughness, dryness, thickening, laxity, telangiectasia, and loss of tensile strength are common features of the aged skin.^15^ The loss in hydration is due to an increase in GAG, which is observed in photoaged skin, whereas, on the contrary, GAG is decreased during chronological aging.^16^ Consequently, the improvement in skin hydration reduces the skin laxity and the skin appears more elastic and stretched.^17^ One of the most vital functions of the skin is to protect the underlying tissues both against dehydration and against environmental risk such as UV exposure, oversheathing or hypothermia, mechanical frictions, and chemical irritants.^18^ The quality of this barrier function is typically assessed by measuring TEWL, which decreases with age, and higher TEWL indicates a more efficient barrier function.^19^ For objective and noninvasive evaluations of skin surface conditions, measurements of hydration, elasticity, turgidity, laxity, and TEWL are widely used.^20^ It is in fact possible to determine the hydration state of the skin surface, that is, water content in the upper portion of the stratum corneum layers. This method detects the efficacy of a cosmetic cream or lotion in terms of enhanced water content in the stratum corneum under constant temperature and moisture conditions.^21^^,^^22^ In contrast, TEWL measurement assesses the barrier function of the stratum corneum by determining the amount of water lost from water-saturated skin tissues as a result of evaporation from the skin surface.^23^ Our retrospective, monocentric, observational study demonstrated an improvement in facial skin hydration, elasticity, and TEWL after 2 months of treatment, proving that the used method is able to efficiently counteract facial aging.

Patient comfort and the absence of untoward effects are absolutely relevant in aesthetic procedures.^24^ Our study confirms the excellent outcome, without major and minor side effects. Only 12.1% of cases reported the presence of small hematomas that disappeared in a few days. Although the injected product was high in concentration (32 mg/mL), the physician judged it easy to inject. Patients were very satisfied at the end of the treatment (87.9%) and the compound's profit evaluated by the physician was optimal in 51.5% of the cases and good in 45.5%. None of the patients expressed negative opinions, and no pain was reported.

The injection of biorevitalizing medical devices in lower impedance sites has some advantages. Among these, the standardization of the injective procedures, and the reduction in pain and intolerance symptoms, because lower impedance areas are quite far from sensitive nerves and blood vessels (100-200 μm), allows diffusion of the compound along the interstitial spaces between lymphatic vessels,^13^ which flow parallel to arterioles, blood vessels, and nerves in the derma. Thus, the product can stimulate cell proliferation in the facial adipose tissue (Bichat fat pad), which is a source of noncommittal staminal cells that differentiate into cutaneous fibroblasts.^25^^,^^26^ The administration of a high-osmolality compound (eg, Profhilo) in lymphatic vessel–enriched areas facilitates the diffusion until the achievement of an osmotic equilibrium. In our hypothesis, in fact, Profhilo attracts water molecules through the lymphatic fenestrated capillary endothelium facilitating the diffusion through the interstitial spaces to achieve the equilibrium condition.

## CONCLUSIONS

In recent years, HA-based fillers are becoming the criterion standard in cosmetic soft tissue and dermal correction. Profhilo represents a good treatment option to restore vitality and turgidity of skin presenting the signs of aging in the absence of intolerance symptoms thanks to its high biocompatibility. The noninvasive injective procedure achieved a good patient compliance with the treatment.

## Figures and Tables

**Table 1:** *Mean* ± *SEM values of facial skin hydration assessed at T_0_, T_1_, and T_2_*

	**Hydration, mean ± SEM**		
	***T*_0_**	***T*_1_**	***T*_2_**
Right	329.9 ± 10.2	348.4 ± 11.3	399.9 ±11.5
Left	333.3 ± 13.5	381.5 ± 13.7	430.9 ± 13.8

**Table 2:** *Mean* ± *SEM values of facial skin elasticity (Young's modulus) assessed at T_0_, T_1_, and T_2_*

	**Elasticity (Young's modulus), mean ± SEM**		
	***T*_0_**	***T*_1_**	***T*_2_**
Right	9.0 ± 0.7	7.9 ± 0.9	7.0 ± 0.5
Left	9.8 ± 0.9	7.8 ± 0.7	6.45 ± 0.7

**Table 3:** *Mean* ± *SEM values of facial skin TEWL assessed at T_0_, T_1_, and T_2_*

	**TEWL, mean ± SEM**		
	***T*_0_**	***T*_1_**	***T*_2_**
Right	10.1 ± 2.5	13.1 ± 2.6	15.1 ± 1.3
Left	11.0 ± 2.4	14.7 ± 2.7	16.2 ± 2.7

TEWL indicates transepidermal water loss.
